# Generation and Auditory Phenotypic Characterization of Prps1 p.Ala87Thr Mouse Knock‐In Model for Human DFNX1 Deafness

**DOI:** 10.1111/cge.14776

**Published:** 2025-06-10

**Authors:** Denise Yan, M'hamed Grati, Rahul Mittal, Yi‐Zhou Quan, Wan Du, Zheng‐Yi Chen, Xue Zhong Liu

**Affiliations:** ^1^ Department of Otolaryngology University of Miami Miller School of Medicine Miami Florida USA; ^2^ Department of Otology and Laryngology Harvard Medical School and Eaton‐Peabody Laboratory, Massachusetts Eye and Ear Infirmary Boston USA; ^3^ John P. Hussman Institute for Human Genomics University of Miami Miami Florida USA

**Keywords:** DFNX1, enzymatic activity, mouse model, progressive hearing loss, *Prps1*varant, spiral ganglion neurons

## Abstract

Variants in the phosphoribosylpyrophosphate synthetase (*PRPS1*) gene have been shown to cause X‐linked nonsyndromic hearing loss (HL) (DFNX1) in humans. A c.259G>A transition in *PRPS1*, which leads to p.Ala87Thr, has been demonstrated to cause HL. The aim of this study was to generate a transgenic knock‐in (KI) mouse with the Prps1 missense variant p.Ala87Thr and to study its impact on the auditory phenotype. Compared to wild‐type (WT) control, transgenic *Prps1* KI mice started to exhibit HL at 32 kHz at 4–12 weeks of age, with HL extending to 8 and 16 kHz by 48 weeks of age. A significant decrease in the number of hair cells and spiral ganglion neuron (SGN) counts was observed at 48 weeks of age in transgenic KI mice. These traits may be associated with the Bak‐dependent mitochondrial apoptosis program, which is triggered by oxidative stress and has been identified as a key mechanism of age‐related HL in C57BL/6J mice. Enzymatic assay showed a significant reduction in Prps1 enzymatic activity in KI compared to WT animals. The Prps1 p.Ala87Thr KI mouse model will serve as a valuable tool for developing therapeutic strategies to mitigate HL associated with *PRPS1* variants.

## Graphical abstract

Our mouse model with a c.259G>A transition in *PRPS1* showed a significant decrease in the number of hair cells and SGN counts at 48 weeks of age and a reduction in Prps1 enzymatic activity in the KI mouse. This model will serve as a valuable tool for developing therapeutic strategies.

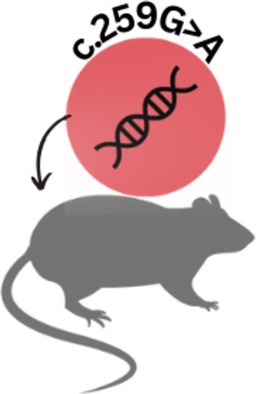

## 1 Introduction

X‐linked deafness is a clinically and genetically heterogeneous disorder that accounts for less than 2% of nonsyndromic hearing loss (NSHL) [1, 2]. To date, seven X‐linked nonsyndromic loci have been discovered, and six X‐linked nonsyndromic genes have been identified, including *PRPS1*, *POU3F4*, *SMPX*, *COL4A6, AIFM1*, and *GPRASP2* (http://hereditaryhearingloss.org) (Accessed 4 Jan 2025). Human phosphoribosylpyrophosphate synthetases (PRS) play an important role in the *de novo* synthesis of purine, pyrimidine, and pyridine nucleotides and the salvage of purine bases [3, 4, 5, 6]. Phosphoribosylpyrophosphate synthetase 1 is most ubiquitously expressed among the three highly conserved PRS genes (*PRPS1*, *PRPS2*, and *PRPS1L1*). PRS‐I, encoded by *PRPS1*, catalyzes the synthesis of phosphoribosyl pyrophosphate (PRPP) from ATP and ribose‐5‐phosphate [7]. DFNX1 (formerly DFN*2*) on Xq22 is caused by **a** loss‐of‐function (LOF) variant in the ***PRPS1*** gene. To date, approximately 29 missense variants in *PRPS1* have been associated with neurological disorders (https://databases.lovd.nl/shared/variants/PRPS1) [8, 9, 10, 11, 12].

These disorders can be caused either by gain of function with increased PRS‐I levels leading to PRS‐I superactivity, or by loss of function with reduced PRS‐I resulting in nonsyndromic X‐linked sensorineural deafness (DFNX1), CMTX5 and Arts syndrome, and retinal dystrophy [13, 14, 15]. All reported disorders have syndromes associated with hearing including prelingual progressive sensorineural hearing loss (HL) and postlingual hearing impairment [13, 16, 17, 18, 19, 20]. Variant screening of the first British American DFN*2* family reported has led to the identification of a G‐to‐A transition at nucleotide position 259, which substitutes Alanine (a nonpolar small hydrophobic amino acid) for Threonine (an uncharged polar larger amino acid) (p.Ala87Thr) [21]. The location of p.Ala87 is at the middle of the β5 strand of PRS‐I enzyme (the second β strand in an anti‐parallel β sheet, also one of end points of flexible ATP‐binding loop). PyMOL predicted that the secondary structure of the antiparallel β sheet may be altered by p.Ala87Thr, which would thus depress the ATP‐binding efficiency [21]. Missense variant p.Ala87Thr results in a loss of PRS‐I activity, as was shown *in silico* by structural analysis and was shown in vitro by enzymatic activity assays in erythrocytes and cultured fibroblasts from patients [21]. The missense variant (rs*180177152*) was absent in different tested populations, with a global minor allele frequency ~ 0/660 (ALFA). In this study, we generated a transgenic knock‐in (KI) mutant mouse with the *Prps1* nonsyndromic HL causative missense variant p.Ala87Thr through injection of recombinant embryonic stem (ES) cells. The widely used C57BL/6 mouse strain was utilized because of its high genetic uniformity, which allows for reliable and reproducible results due to minimal genetic variability.

## 2 Materials and Methods

### 2.1 Gene Targeting and Generation of Prps1 Transgenic Mouse

**FIGURE 1:**
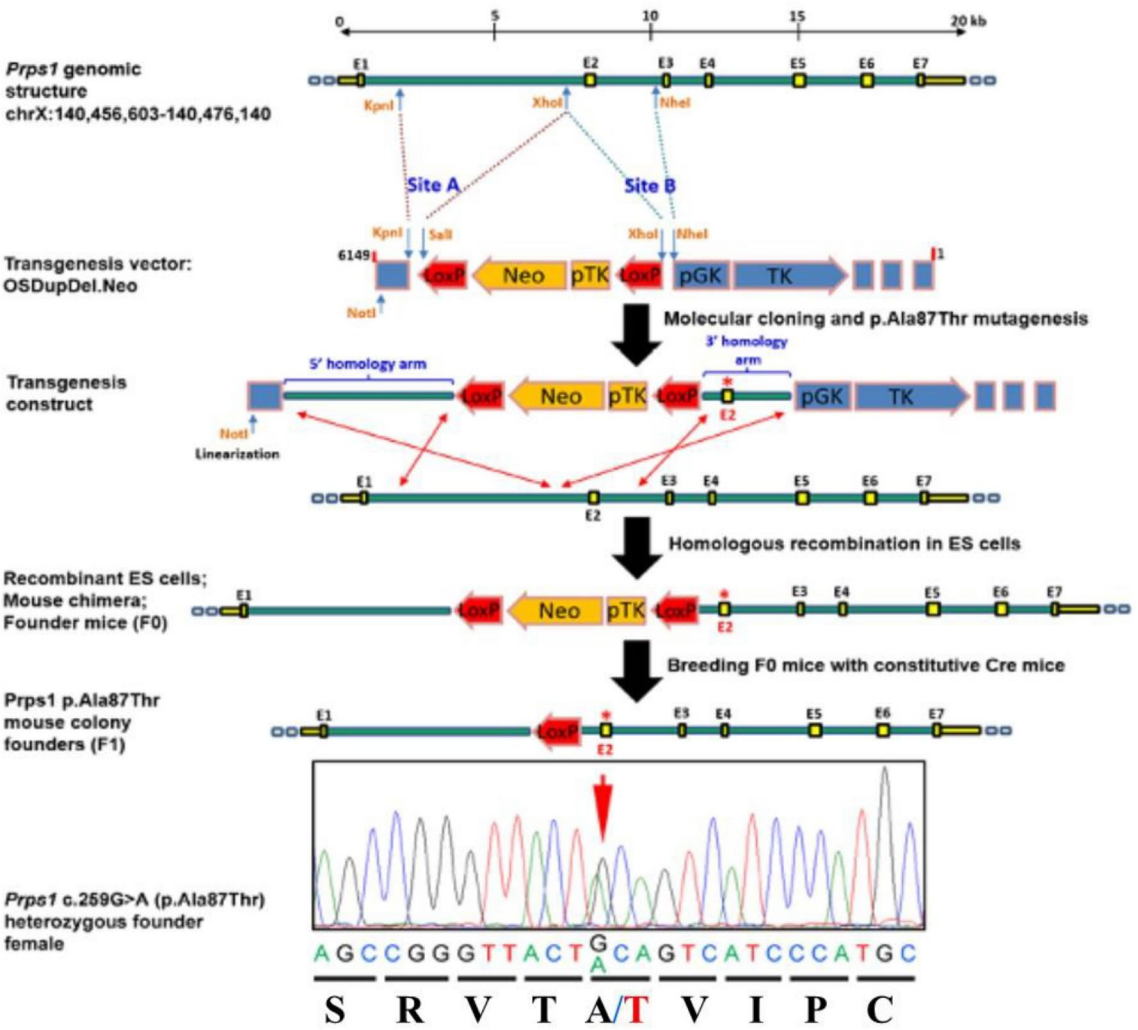
The *Prps1* KI mouse is a model for human non‐syndromic X‐linked sensorineural deafness DFNX1. (A) Schematic of the engineered targeting construct for transgenic *Prps1* KI mouse for mutation c.259G>A causing p.Ala87Thr; (B) Electrophorogram of the genomic DNA sequence of heterozygous *Prps1* ^*KI/+*^ founder female mouse.

The *Prps1* transgenic mice were generated at the University of Miami transgenic core facility led by Dr. Yincai Wang (Figure 1). The first step consisted of cloning *Prps1* 5′–and 3′–homology arms into OSDupDel.Neo transgenesis vector (Open Biosystems, catalog# MES3974). *Prps1* genomic restriction map was constructed on C57BL/6J genomic DNA obtained from UCSC Genome Browser Dec. 2011 (GRCm38/mm10) assembly (https://genome.ucsc.edu/). Both homology arms were amplified using High Fidelity *Taq* DNA polymerase (Invitrogen) on C57BL/6J female genomic DNA template; the c.259G>A (p.Ala87Thr) was placed within the 3′–homology arm. The homology arms were subsequently sub‐cloned into pCR.TOPO T/A cloning vector (Invitrogen), and one positive clone for each of the inserts was selected for further processing after full‐length insert sequence by Sanger sequencing. Clone containing 3′–homology arm was subjected to site‐directed mutagenesis aiming to introduce the *Prps1* c.259G>A variant in exon 2. After mutagenesis reaction using QuikChange II XL Site‐Directed Mutagenesis Kit (Agilent Technologies) and bacteria transformation, 50 clones in total were isolated and sequenced to search for the desired mutation, and the entire sequence of one positive construct was identified using Sanger sequencing. We first directionally cloned c.259G>A mutated 3′–homology arm into XhoI‐NheI restriction sites into OSDupDel.Neo vector, followed by inserting the 5′–homology arm into KpnI‐XhoI restriction sites. Finally, the final mutagenesis construct was linearized using NotI enzyme and agarose gel‐tested, and subsequently purified. The linearized construct was then electroporated into 129/SvEv ES cells. Recombinant ES cells were screened by long‐range PCR of genomic regions covering both 3′ and 5′ recombination arms, using primers on the reporter cassette and bordering the recombination arms. Two recombinant ES cell clones were then transplanted into pseudo‐pregnant C57BL/6J females. Chimeras were obtained, backcrossed to C57BL/6J mates, and germline transmission was tested through F0 litter genotyping using long‐range PCR. One founder female from each of the transplanted ES cell clones was used to generate distinct colonies. Founder females, containing floxed reporter cassette and *Prps1* c.259G>A variant, were crossed to transgenic males (N11F5 on C57BL/6J background) ubiquitously expressing recombinase Cre under the control of human cytomegalovirus (CMV) promoter (Mouse–B6.C‐Tg (CMV‐cre)‐1cgn/J (JAX stock# 006054)). Heterozygous female carriers were identified by PCR of intron 1 region encompassing a residual LoxP site in the mutant allele and confirmed by an additional PCR of exon 2 and sequencing of *Prps1* c.259G>A variant. The heterozygous female mutant with hemizygous mutant crossing scheme was subsequently used for expanding the colony.

### 2.2 Auditory Evaluations of Mice

Mice were anesthetized using ketamine/xylazine and positioned in a sound‐insulating chamber to minimize external noise interference. Their body temperature was continuously monitored and maintained at ~37°C to prevent hypothermia‐related auditory variability. The Smart EP Universal Smart Box (Intelligent Hearing Systems, Miami, FL) was used for auditory brainstem response (ABR) testing. The stimuli consisted of 0.1 ms duration pure tone pips presented at 8, 16, and 24 kHz. The stimulus intensity ranged from 90 dB SPL down to 10 dB SPL, in 5 dB decrements, to determine the hearing threshold for each frequency. Each frequency and amplitude condition was averaged over 600 sweeps. ABR thresholds were determined by identifying the lowest intensity at which a recognizable ABR pattern was observed, defined by the presence of at least two consistent peaks above the baseline as described by Noben‐Trauth et al. (2010) [22]. The data were independently evaluated and validated by at least two investigators who were blinded to the experimental groups.

### 2.3 Histopathological Examination

Animals were euthanized at 48 weeks for histological evaluation. Subsequently, the temporal bones were harvested, and the tympanic bullae were quickly dissected from the temporal bones and prepared for microscopic examination. After fixation in 10% buffered formalin solution for 48 h, the specimens were decalcified using 10% ethylenediaminetetraacetic acid (EDTA) and then embedded in paraffin and sectioned (Leica microtome, Germany) followed by staining with hematoxylin and eosin as described in previous studies [23]. For hair cell immunofluorescence staining, MYO7A antibody (1:500, Proteus Biosciences 25‐6790); Alexa Fluor 594 phalloidin (1:500, Thermo Fisher), and Alexa Fluor 488 secondary antibody (1:1000, Invitrogen) were used.

### 2.4 Prps1 Activity Assay

Blood was collected from WT and Prps1 KI mice at 48 weeks of age, followed by the separation of peripheral blood mononuclear cells (PBMCs) and erythrocytes. The red cells were washed three times in phosphate‐buffered saline (PBS), packed, and stored at −20°C until analysis. On the day of the assay, samples were thawed and treated with activated charcoal (3 mg/mL) for 15 min at 4°C to remove potential interfering substances. After centrifugation, the supernatant was collected for Prps1 activity assessment. The enzymatic assay for Prps1 activity was based on the conversion of ribose‐5‐phosphate and ATP to phosphoribosyl pyrophosphate (PRPP) and AMP, catalyzed by Prps1. The reaction mixture contained Tris–HCl buffer (pH 7.4), MgCl_2_, ATP, and ribose‐5‐phosphate, with the reaction initiated by the addition of hemolysates. Following incubation at 37°C for a fixed time interval, the reaction was terminated by heat inactivation. To ensure specificity and accuracy in measuring Prps1 activity, high‐performance liquid chromatography (HPLC) was employed as described in detail in previous studies [24, 25]. The generated AMP was separated using a reverse‐phase column with UV detection at 254 nm. Isocratic elution was performed using a phosphate buffer and acetonitrile mobile phase, optimizing the resolution of AMP from other nucleotides. The retention time and peak area of AMP were used to determine enzymatic activity, with standard curves constructed using known AMP concentrations for precise quantification. The hemoglobin content of each hemolysate was determined using the Drabkin method and served as a normalization factor. Additionally, Prps1 activity was measured in urine samples from WT and Prps1 KI mice at 48 weeks of age, with creatinine levels used for normalization. Prps1 activity was expressed as the concentration of AMP generated per time unit and per mg of hemoglobin in the hemolysate or per mg of creatinine in the urine samples.

### 2.5 Statistic Analysis

2.5.1. For hearing testing, statistical analysis was performed with one‐way ANOVA followed by Bonferroni adjustment as the post hoc method, using SPSS 12 software. The results were considered significant at *p* < 0.05, and the data are presented as mean ± SEM. A total of 6–8 WT or Prps1^−/−^ mice were assessed at different ages.

2.5.2. For quantitation of spiral ganglion neurons in the Prps1 KI mice and determination of the Prps1 activity, Mann–Whitney U test was used. **P**‐values of less than 0.05 were considered to indicate significance. The results were statistically analyzed using SPSS ver. 21.0 (IBM, Armonk, NY, USA). Ten to 12 cochlea from control or mutant mice were used.

2.5.3. For hair cell count (Figures 3 and 4). The Prism 10 statistical package (GraphPad Software Inc) was used in data processing. To count HCs WT vs. KI, multiple regions from the apex (75–100% of the length of cochlear duct from the hook), mid (50–75% of the length of cochlear duct from the hook), and base (25–50% of the length of cochlear duct from the hook) were included. The HC density was calculated as the average number of HCs per 100 μm for each cochlea. Controls were cochleae from WT mice. Multiple cochleae were used for each counting and in the statistical analysis. Data were presented as mean ± SEM. ANOVA analysis with Sidak's multiple comparisons test was used to compare HCs in the apex, middle, and basal turn of the cochlear samples, respectively (*p* < 0.05 was considered significant).

## 3 Results

### 3.1 Description of the Overall Health Status of Prps1 Transgenic KI Mice

Compared to their WT littermates, *Prps1* KI hemizygous male and homozygous female mice had a normal appearance and had a normal development and growth rate. They also had a very similar fur color and density as WT mice. They had normal vision, sexuality, and overall activity/behavior. They did not present signs of vestibular dysfunction, such as circling, head bobbing, or any other characteristic balance defects. No neurological abnormalities were observed as they exhibited normal posture, gait, righting reflex, and hindlimb extension, with no signs of motor dysfunction. Their inner ears developed normally with comparable gross size to their littermate controls.

### 3.2 Prps1 KI Mice Show Mild Progressive Hearing Loss

**FIGURE 2:**
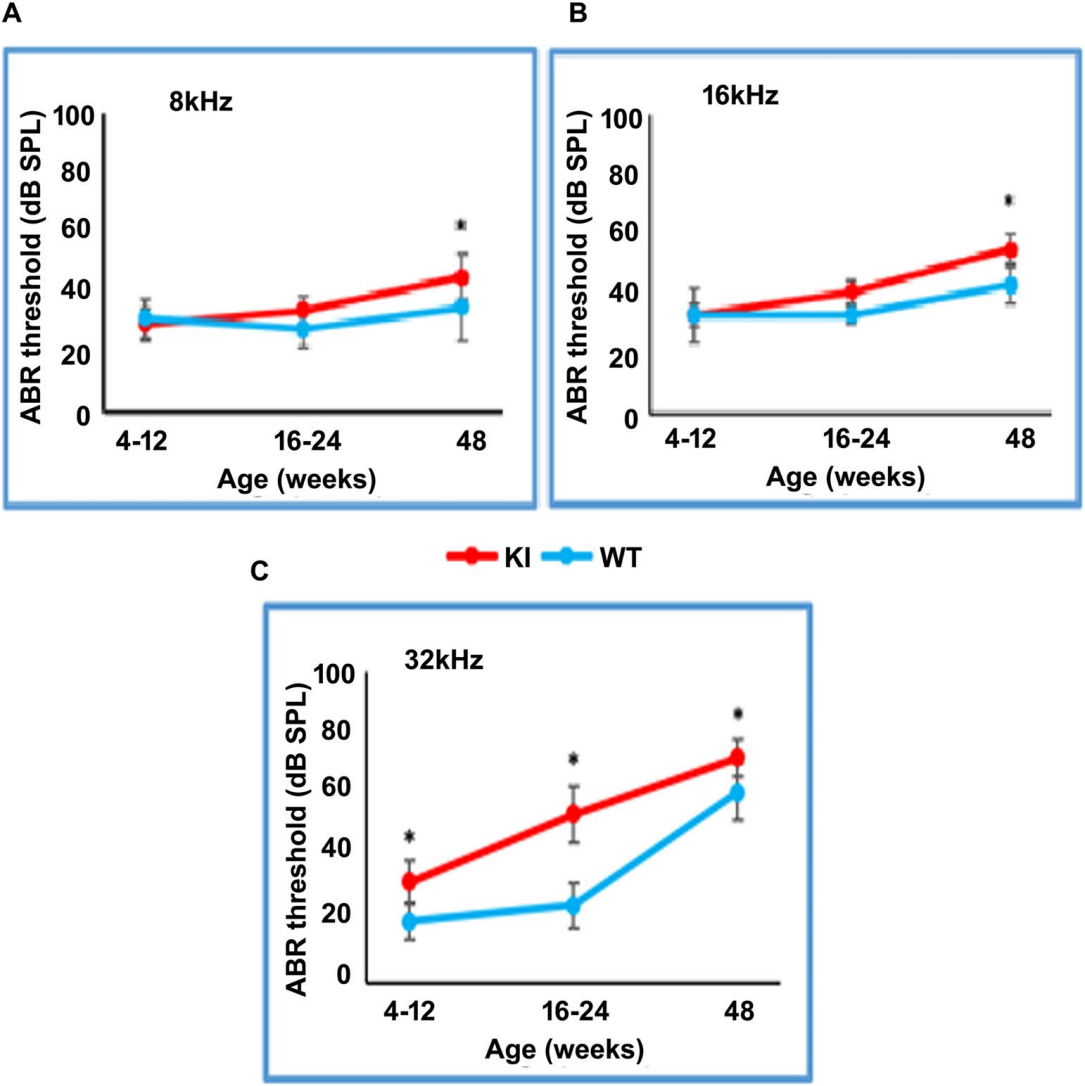
Auditory evaluation of *Prps1* KI mice. Auditory brainstem recording thresholds of pure tone stimuli at 8 kHz (A), 16 kHz (B), and 32 kHz (C) kHz of mutant hemizygous males and homozygous females were evaluated and averaged for ages 4–12, 16–24, and 48 weeks. Data collected for 4–12 weeks of age were extrapolated from ABR measurements on transgenic *Prps1* KI mice (18 hemizygous males and three homozygous females), seven wild‐type males, and seven wild‐type females. Data plotted for 16–24 weeks of age were extrapolated from ABR measurements on transgenic *Prps1* KI mice (12 hemizygous males and 10 homozygous females), seven wild‐type males, and nine wild‐type females. Data presented for 48 weeks of age were extrapolated from ABR measurements on 16 hemizygous males, 8 homozygous females, 9 wild‐type males, and 8 wild‐type females. *p < 0.05.

We measured ABRs to pure tone stimuli in *Prps1* KI mice (average of 20 with a male to female ratio of ~1/1) and on a comparable number of WT littermates starting around 4 weeks of age and up to 48 weeks of age, using pure tone stimuli 8, 16, and 32 kHz (Figure 2A–C). At 4–12 weeks of age, the KI mice presented comparable hearing thresholds to their littermate/age‐matched WT control mice at 8 and 16 kHz (Figure 2A,B). However, a small but significant increase in auditory thresholds was detected at 32 kHz high frequencies (average of 10–15 dB SPL; *p* < 0.05, Figure 2C). At 16–24 weeks of age, the ABR thresholds of the *Prps1* KI mice were further increased at 32 kHz compared to age‐matched WT animals (Figure 2C). At this age, there was a slight increase in the ABR thresholds in the KI mice at 8 and 16 kHz that were not statistically significant compared to WT control mice (Figure 2B). By 48 weeks of age, significant HL was detected at all three tested frequencies in the *Prps1* KI mice, supporting progressive HL in the *Prps1* KI mice (Figure 2A–C).

### 3.3 Loss of Hair Cells and Spiral Ganglion Neurons in the Prps1 KI Mice

**FIGURE 3:**
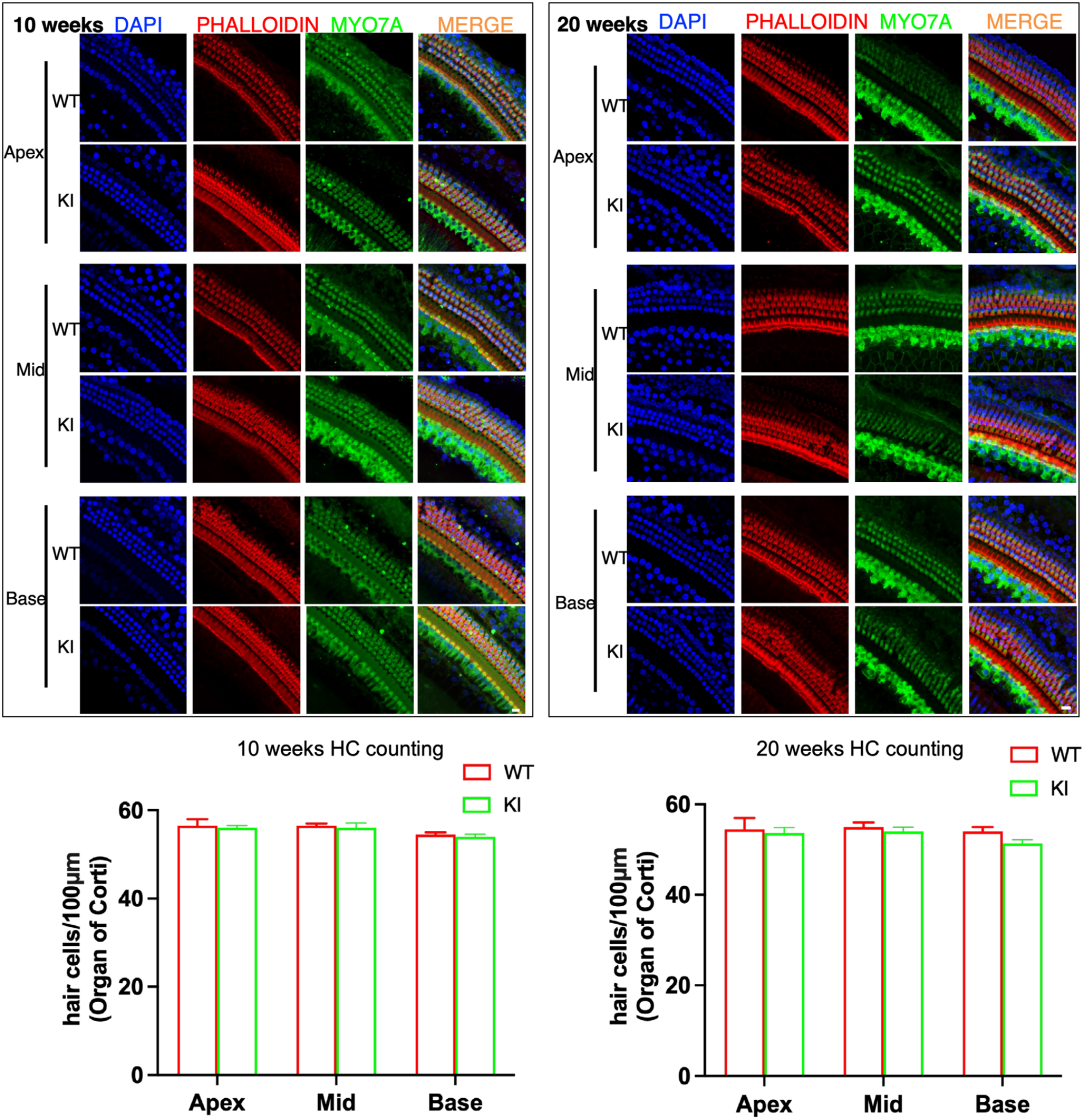
Whole mount immunostaining. Hair cell staining of wild‐type and *Prps1* KI mice at 10 and 20 weeks of age. We did not detect any significant difference in the number of HCs between mutant and controls at 10 and 20 weeks.

**FIGURE 4:**
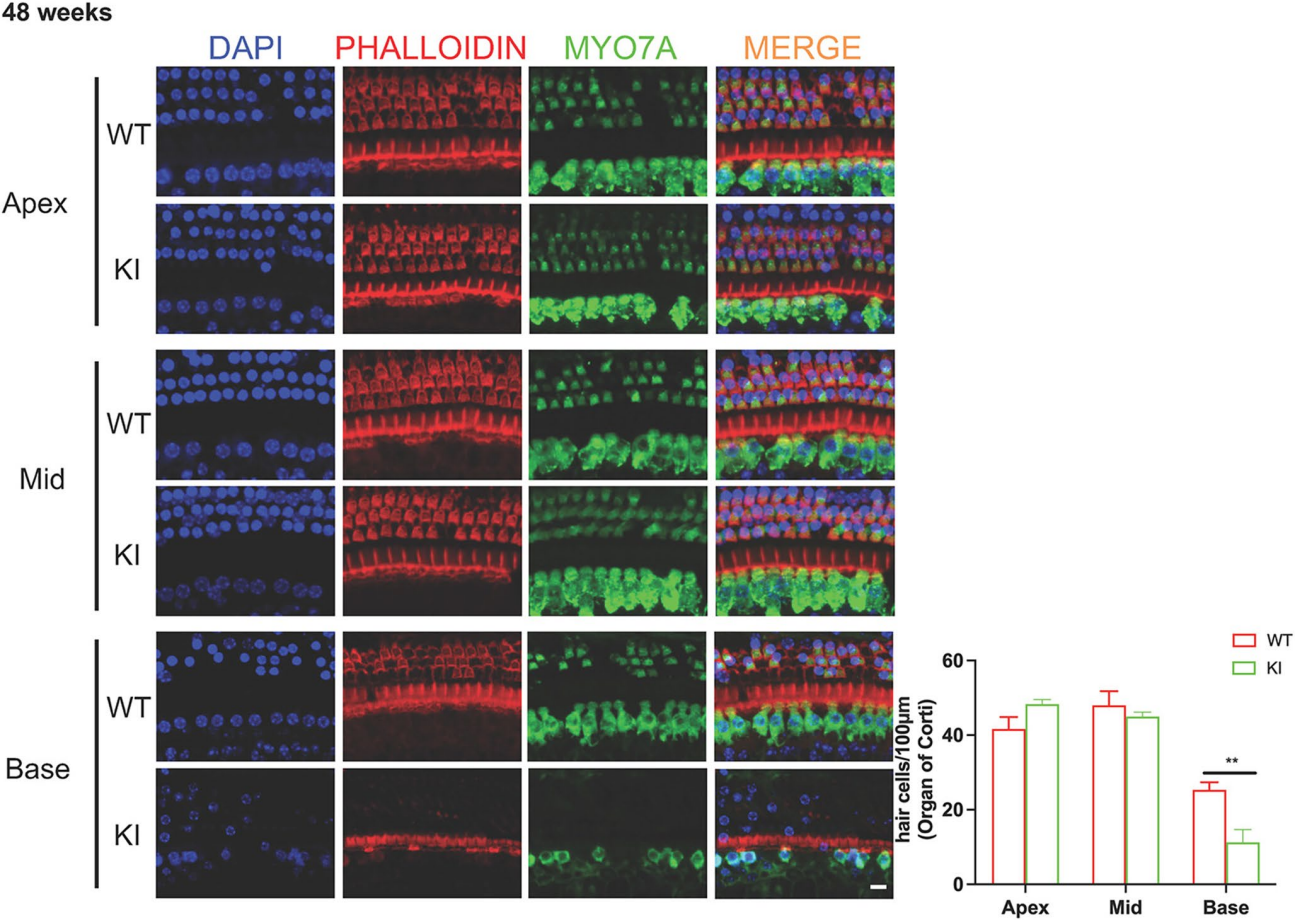
Immunofluorescence staining. Hair cell staining of wild‐type and *Prps1* KI mice at 48 weeks of age. The significant hair cell number difference was detected in the basal turn WT vs. KI groups at 48 weeks. Quantification and comparison showed significantly more MYO7A+ Hair Cells in the basal turn of the cochlear samples (WT vs. KI) at 48 weeks. ****p < 0.0001, two‐way ANOVA with multiple comparisons. Error bar, mean ± SEM; *n* = 2–4 in each group. Scale bars: 10 μm. The significant hair cell number difference was detected in the basal turn WT vs. KI groups at 48 weeks. We did not observe any significant difference in the number of HC between mutants and controls at 10 and 20 weeks.

**FIGURE 5:**
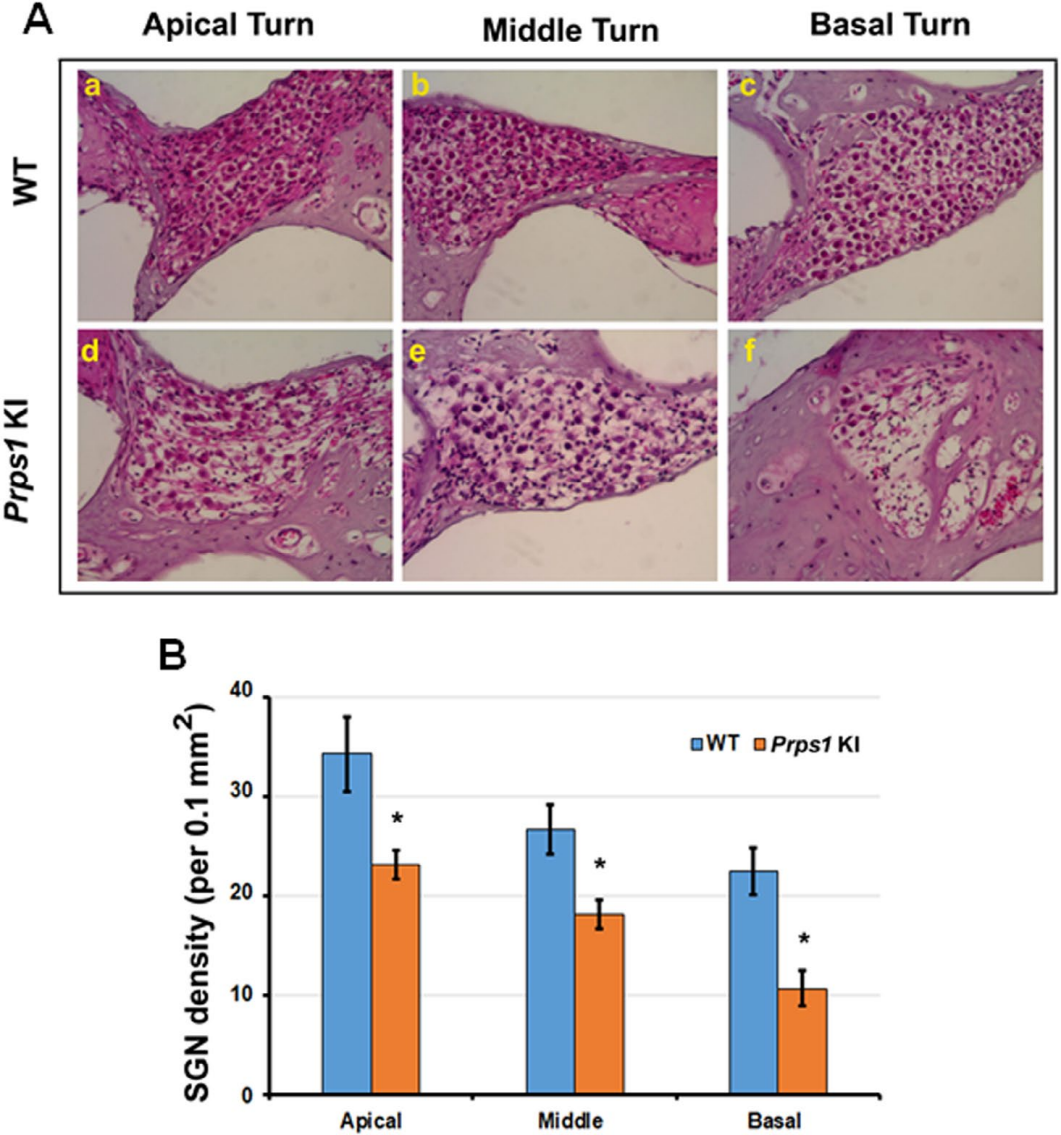
Histopathological examination of cochlea from wild‐type and *Prps1* KI mice. Cochlea were harvested from wild‐type and *Prps1* KI mice and subjected to histopathological examination. We observed degeneration of spiral ganglion neurons (SGNs) in apical (Figure 5A–d), middle (Figure 5A–e) and basal (Figure 5–f) turns in cochlea of *Prps1* KI mice compared to wild‐type animals (Figure 5‐a,b,c). Quantitation of the data revealed that there was significant loss of SGNs in cochlea of *Prps1* KI mice compared to wild‐type animals (B). *p < 0.001 Scale bars: 10 μm.

To understand the inner ear phenotype of the *Prps1* KI mice, we performed immunohistochemistry to study hair cells. Between 10 and 20 weeks of age, we detected no change in hair cell number in the *Prps1* KI mice (Figure 3). By 48 weeks of age, however, a significant decrease in the number of cochlear inner and outer hair cells (OHCs) was detected in the basal turn of the cochlea for *Prps1* KI mice (Figure 4), indicating that the progressive nature of the HL in the *Prps*1 mutant mice may result from progressive cell death of cochlear hair cells. Comparison of the percentage of OHC and inner hair cell (IHC) loss in WT and mutant mouse (KI) models from the apex to the base as a function of age is shown in Table 1A,B. We performed histopathological examination of the cochlear tissues of *Prps1* KI and WT mice at different time points. There was a large number of empty spaces scattered throughout Rosenthal's canal, indicating loss of spiral ganglion neurons (SGNs) in the apical, middle, and basal turns of the cochlea in *Prps1* KI mice compared to WT animals at 48 weeks of age (Figure 5A). Quantitation of the SGN showed that there was a significant decrease in the SGN density in *Prps1* KI mice at 48 weeks of age (Figure 5B). The data support the requirement of Prps1 for the age‐dependent survival of auditory hair cells and SGNs.

**TABLE 1:** Table comparing the percentage of outer hair cell (OHC) and inner hair cell (IHC) loss in wild‐type (WT) and mutant mouse (KI) model from the apex to the base as a function of age.

A Percentage of OHC loss		
10 weeks	Mean (KI)	Mean (WT)
Apex	0%	1%
Mid	1%	0%
Base	1%	0%
20 weeks	Mean (KI)	Mean (WT)
Apex	0%	0%
Mid	6%	1%
Base	7%	1%
48 weeks	Mean (KI)	Mean (WT)
Apex	12%	11%
Mid	10%	10%
Base	84%	54%

B Percentage of IHC loss		
10 weeks	Mean (KI)	Mean (WT)
Apex	0%	0%
Mid	0%	0%
Base	0%	0%
20 weeks	Mean (KI)	Mean (WT)
Apex	0%	0%
Mid	0%	0%
Base	1%	0%
48 weeks	Mean (KI)	Mean (WT)
Apex	6%	8%
Mid	5%	5%
Base	23%	18%

### 3.4 Prps1 KI Mice Exhibit Significant Reduction in Enzymatic Activity

**FIGURE 6:**
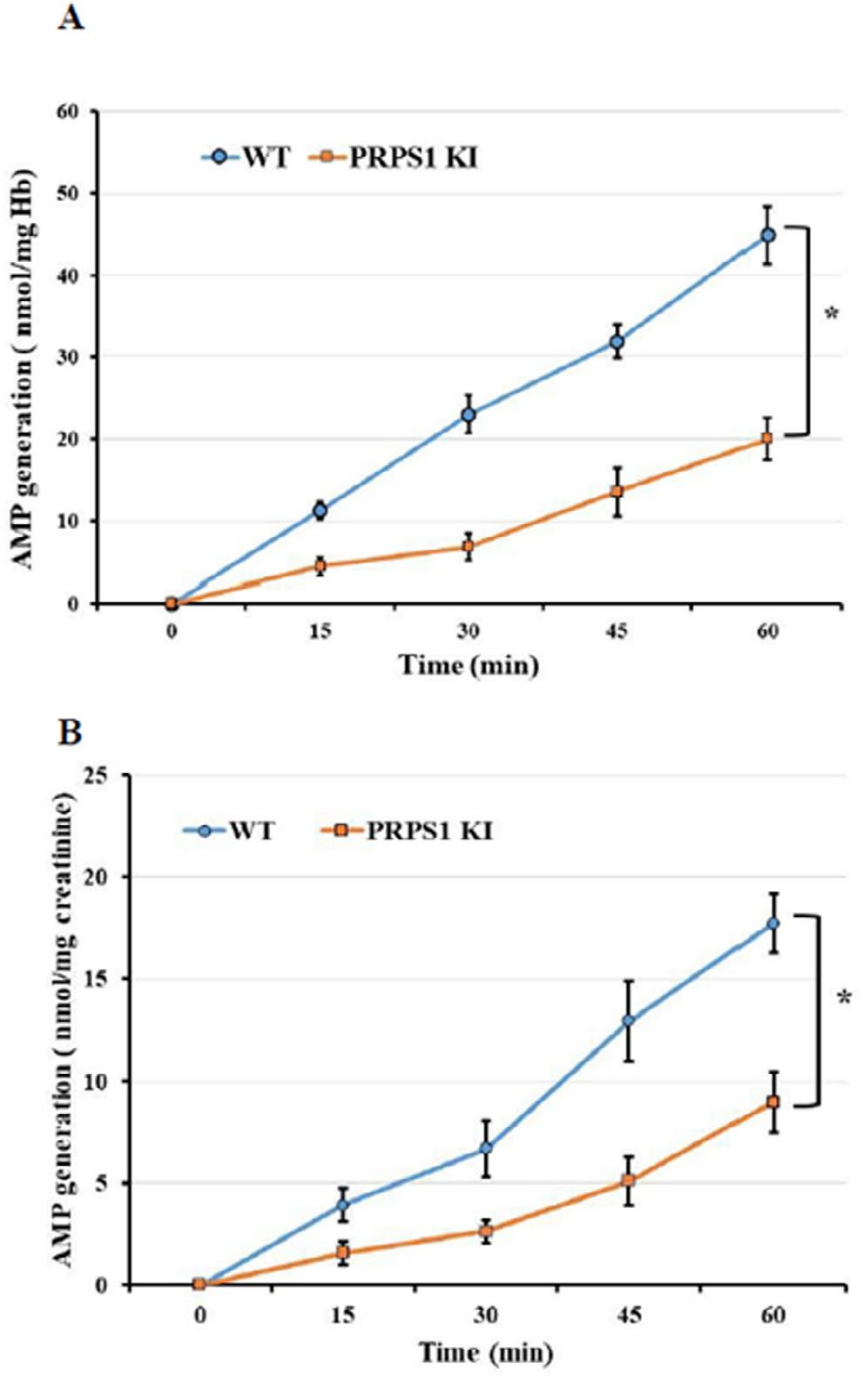
Functional characterization of Prps1 activity in transgenic mice. Prps1 activity in erythrocytes (**A**) and urine (**B**) of wild‐type and transgenic mice at 48 weeks of age was determined by measuring the accumulation of AMP by HPLC. *p < 0.01.

We determined PRPS1 enzymatic activity in erythrocytes and urine of WT and *Prps1* KI mice by assessing the adenosine monophosphate (AMP) generation at different time points using HPLC. There was a statistically significant difference in PRPS1 enzymatic activity at all time points tested in erythrocytes from *Prps1* KI mice compared to wild‐type (WT) animals (*p* < 0.01) (Figure 6A). Similar decreases in PRPS1 enzymatic activity were observed in the urine samples of *Prps1* KI mice compared to WT animals (*p* < 0.01) (Figure 6B).

## 4 Discussion

Consistent with the milder phenotype, the mutant p.Ala87Thr KI mice have overall normal cochlear morphology up to 10 to 20 weeks of age. At 48 weeks of age, the reduction in the number of cochlear hair cells (HCS) was mainly detected in the basal turn, whereas the loss of SGNs was detected throughout the cochlea. In the assessment of the hearing thresholds, we observed that the transgenic hemizygous males and homozygous female mice had more severe HL in high frequencies as compared to low and middle frequencies. The Prps1 p.Ala87Thr KI mice also displayed at the 48 weeks of age, reduced Prps1 enzymatic activity in erythrocytes and urine compared to WT littermate control animals. The C57BL/6 mouse strain is known to exhibit a natural progression of cochlear hair cells (HCS) degeneration, starting in the basal turn of the cochlea, progressing to the middle and apical turns with increasing age, often accompanied with a subsequent decline in IHC functional integrity and a gradual loss of SGNs in the same region; this pattern leads to a progressive high‐frequency HL [26]. Thus, a confounding factor due to our choice of C57BL/6 mouse strain for our knock in model generation could have impacted the observable characteristics of the p.Ala87Thr mutation, potentially obscuring the effects of the targeted gene modification itself. Age‐related hair cell loss in C57BL/6J has been reported to progress along a base‐to‐apex gradient with more loss observed at 26 months compared to 22 months [26]. While the loss of hair cells is likely to contribute to HL, the degeneration of SGNs is more closely correlated with HL observed across different frequencies (Figure 2C). In agreement with earlier studies that have demonstrated that degeneration of SGNs has been associated with HL in mouse models, our study highlights age‐related SGN loss in C57BL/6J mouse strain is being a significant contributor to age‐related hearing impairment. *Saposin B*‐deficient mice exhibit a similar pattern of progressive HL due to degeneration of SGNs despite having normal number of OHCs [27]. We found a reduction of PRPS1 enzymatic activity in erythrocytes and urine of Prps1 p.Ala87Thr KI mouse model in function of AMP generation, that has previously been reported in humans with a LOF variant in PRPS1 [10, 21]. To the best of our knowledge, this is the first report of decreased AMP generation in mouse erythrocytes and urine. Taken together, we show that a reduction of PRPS1 activity is associated with the loss of cochlear HCs, SGNs, as well as a concomitant loss of hearing in mice. Although our data support the role of PRPS1 in the survival of hair cells and SGNs that is age‐dependent, it has also been established that in response to increased oxidative DNA damage in the cochlea of C57BL/6J aged mice, p53 translocates to mitochondria and activates Bak, leading to Bak‐mediated apoptosis and eventually to cochlear cell death, and Bak deficiency prevents apoptotic cell death [28, 29, 30, 31]. While human DFNX1 patients are characterized by postlingual progressive nonsyndromic HL, inter‐ and intra‐ familial phenotypic variability of clinical expression has also been reported [9]. The clinical feature manifestations may be due to several biological factors, including (a) functional redundancy and compensation among PRS isoforms; (b) differences in the rate‐limiting enzyme, PRPP amidotransferase (PPAT) expression levels; (c) skewed X‐chromosome inactivation in females or; (d) effect of modifier *loci*. In the British‐American family reported by Tyson et al. (1996) [18], the HL was congenital and profound. Hemizygous male patients exhibited flat shaped audio profiles, and obligate carrier females showed mild to moderate HL affecting the high frequencies. In this family, we identified a G‐to‐A transition at nucleotide position 259 that replaces a nonpolar small hydrophobic amino acid—alanine—with an uncharged, polar, larger amino acid—threonine (A87T) [21]. Here, we report the generation and characterization of the Prps1 p.Ala87Thr KI mice. This KI transgenic mouse model clearly illustrates the inner ear pathology caused by the variant in hair cell and SGN loss with the phenotype that closely mimics human clinical conditions including progressive delayed onset HL. However, the “genetic background effect” of C57BL/6, used for the generation of the KI mouse model may have influenced the phenotype of the engineered *Prps1* nonsyndromic HL causative mutation, making it difficult to accurately interpret the results of the study.

Besides the PRPS1 pathway, purine nucleotides can be generated through an alternative pathway that utilizes S‐adenosylmethionine (SAM). Therefore, it will be worthwhile in future studies to explore the effect of SAM supplementation on the amelioration of auditory symptoms utilizing this transgenic mouse model. In addition, this KI mouse model can be used to determine the efficacy of genome editing techniques in rescuing the function of Prps1. The availability of new therapeutic strategies for the management of HL will lead to an improved quality of life for many hearing‐impaired individuals and their families.

## Author Contributions

D.Y., R.M., M.G., Y.Z.Q., W.D. contributed to methodology, acquisition of data, writing of original draft; D.Y., R.M., Y.Z.Q., W.D. performed data acquisition, writing of original draft; D.Y., R.M., and Z.‐Y.C. contributed to content revision; D.Y., R.M., and Z.‐Y.C. contributed to conceptualization and content revision; X.Z.L. contributed to conceptualization, design, and content revision. All authors approved the final version for publication.

## Ethics Statement

All procedures were approved by the University of Miami Institutional Animal Care and Use Committee following the National Institutes of Health guidelines.

## Conflicts of Interest

The authors declare no conflicts of interest.

## Peer Review

The peer review history for this article is available at https://www.webofscience.com/api/gateway/wos/peer‐review/10.1111/cge.14776.

## Data Availability

The data that support the findings of this study are available from the corresponding author upon reasonable request.
